# Clinical Lectures on Diseases of the Eye

**Published:** 1866-01

**Authors:** E. L. Holmes

**Affiliations:** Lecturer on Diseases of the Eye in Rush Medical College, and Surgeon to the Chicago Charitable Eye and Ear Infirmary


### CHICAGO MEDICAL JOURNAL.

Vol. XXIII. JANUARY, 1866. No. 1.

### ORIGINAL CONTRIBUTIONS.

*Clinical Lectures on Diseases of the Eye.* By E. L. Holmes, M. D., Lecturer on Diseases of the Eye in Rush Medical College, and Surgeon to the Chicago Charitable Eye and Ear Infirmary.

### THE ACCOMMODATION OF THE EYE TO DISTINCT VISION AT DIFFERENT DISTANCES.

Gentlemen,—Before discussing the anomalies of accommodation, it is well to examine the normal formation of the eye with some principles of optics especially connected with vision.

An eye is said to be normal in formation, when its refracting media possess such a degree of convexity, with a power of accommodation, and with the distances between the retina, lens and cornea such, that the rays of light may be brought to a focus upon the retina from objects at any distance more than five inches from the cornea.

Although the eye may thus adjust its focus for objects within the limits described, distinct perception of these objects depends upon the sensitiveness of the retina, which, like other nervous structures, requires a certain amount of impression to produce sensation. As the nerves of the skin, for instance, fail to perceive slight changes in degrees of heat, or in force of galvanic influence, so the retina fails to perceive objects, either when they are too dimly lighted, or so far away, that the images on the retina are too small. The most sensitive portion of the skin perceives the contact of the two points of a pair of compasses as two distinct impressions, only when the points are separated from each other more than seven or eight tenths of a line. The retina is vastly more sensitive since it can distinguish two luminous points, as separate objects, when they form images on the retina less than one three-thousandth of an inch apart. It may be here stated, that suitable exercise of the eye upon very minute near objects, or very distant objects, increases the sensitiveness of the retina.

Objects too brilliantly illuminated, or presenting too great contrasts of light and shade, are not easily distinguished, even when the focus of the eye is properly adjusted. As illustrations, may be mentioned the difficulty, if not impossibility, of distinguishing the form of the sun's disk in a partial eclipse; and also the discomfort experienced in looking at objects while facing a bright window.

In proportion as the sensitiveness of the retina or optic nerve is impaired by disease, the eye fails to perceive objects; and yet in some diseases of the nerve, vision is quite good, if the images on the retina are increased in size by placing the objects nearer, or if objects are illuminated more brightly than usual. Many patients with amaurosis of slight degree are thus able to perform quite readily the ordinary duties of life. Such patients often compensate for the abnormal condition of the nervous structure by the use of double convex lenses, which produce larger images on the retina.

There are a few facts regarding the passage of light through convex lenses, which should be noticed in connection with the subject of accommodation. Rays of light, as we have seen, from very distant objects, possess so slight a degree of divergency, that they may be regarded for all practical purposes as parallel. Such rays form images on a screen at a distance behind a lens equal to the radius of its curvature. When distant objects are moved towards the eye, within certain limits, the rays possess so little divergency, that they may still be regarded as parallel, since very distinct images will be produced at the focus of parallel rays.

Hence the images of two objects of suitable size, one placed at seventy and the other at fifty feet from a photographer's camèra, are both found to be distinct on the ground glass screen. Hence, also, remote objects at different distances may be seen distinctly with no perceptible effort of the muscles of accommodation.

The rays of light from *near* objects, however, as they are brought nearer the eye, become so rapidly more divergent, that comparatively slight changes of distance require great effort on the part of the adjusting apparatus.

The internal recti muscles perform an important part in connection with the act of accommodation. While in rotating the eyes from one side to the other, each internal rectus acts with the external rectus of the other eye. In accommodating the eyes to near objects, the two internal recti act together, rendering the visual axes convergent, or, in other words, "fixing" the eyes upon one point. These muscles, however, cannot, except in abnormal conditions of the eye, "fix" both eyes upon one point nearer the cornea than five inches. It should be remarked, that the degree of convergence of the visual axes increases so slightly, as very remote objects are brought nearer the eye, that we have scarcely any perception of muscular effort in the internal recti for changes in distance of remote objects; while great muscular effort is required when *near* objects are brought nearer the eye.

As the iris, by the contraction of the pupil in looking at near objects, excludes from the eye the more divergent portion of the rays which fall on the cornea, it can be readily understood, from what has been said above, that the rays which pass through the contracted pupil possess so little divergency, that they form distinct images on the retina, even when the eye (lens) is not accurately adjusted for these objects.

Upon the same principle, a minute aperture in a thin disk of metal, placed in front of the pupil, and as near the cornea as possible, enables us to distinguish objects distinctly with scarcely any aid of the adjusting apparatus. Thus the iris not only regulates the amount of light which enters the eye, but it also serves as a diaphragm in excluding the more divergent rays of light.

The distribution of nerves to the parts of the eye upon which the power of accommodation depends, is quite complex, and not altogether well understood. Upon branches of the fifth pair of nerves depends sensation in these organs of the eye. From the third pair are supplied the nerves of motion in the internal recti and the annular muscular fibres of the iris, and possibly of the ciliary band. From the sympathetic are probably supplied the nerves of motion to the radial fibres of the iris and ciliary muscle.

For further development of these interesting subjects, especially for a description of the manner of measuring and expressing by figures the extent of accommodation, I must refer you in particular to the works of Donders and Jaeger.

